# Impact of Harvest Periods on the Physicochemical and Flavour Characteristics of Sichuan Pepper (*Zanthoxylum bungeanum* Maxim)

**DOI:** 10.3390/foods14071155

**Published:** 2025-03-26

**Authors:** Lian He, Yuwen Yi, Hongfeng Jia, Chengjian Xu, Mingfeng Qiao, Xuemei Cai, Sze Ying Leong, Nallammai Singaram, Sook Wah Chan, Hua Peng

**Affiliations:** 1 College of Culinary and Food Science Engineering, Sichuan Tourism University, 459 Hongling Road, Longquanyi District, Chengdu 610100, China; ai_helian@163.com (L.H.); helloxj2000@gmail.com (C.X.); 2 School of Biosciences, Faculty of Health and Medical Sciences, Taylor’s University, Subang Jaya 47500, Selangor, Malaysia; szeying.leong@taylors.edu.my (S.Y.L.); nallammai.singaram@taylors.edu.my (N.S.); 3 Cuisine Science Key Laboratory of Sichuan Province, Sichuan Tourism University, 459 Hongling Road, Longquanyi District, Chengdu 610100, China; jeff-800911@126.com (Y.Y.); 0000374@sctu.edu.cn (H.J.); mfqiao@163.com (M.Q.); cxm121517@163.com (X.C.); 4 Food Security & Nutrition Impact Lab, Taylor’s University, Subang Jaya 47500, Selangor, Malaysia; 5 Clean Technology Impact Lab, Taylor’s University, Subang Jaya 47500, Selangor, Malaysia; 6 Research Center for Tourism Agriculture Development, Sichuan Tourism University, Chengdu 610100, China

**Keywords:** Sichuan pepper, 24 solar terms, harvest periods, physicochemical properties, flavour characteristics

## Abstract

Sichuan pepper is known for its unique aroma and tingling, numbing sensation, making it a key ingredient in Sichuan cuisine. This study explored the effect of harvest periods on the quality of Sichuan pepper at five selected harvest periods (LSA (early harvest), LSB, LSC, LSD, and LSE (late harvest)) along the 24 solar terms in the traditional Chinese lunar calendar. Apart from evaluating their physicochemical and volatile profiles, the growth characteristics, polyphenol and flavonoid contents, antioxidant properties, and free amino acid and other nutrient concentrations in these peppers were also analysed. Results showed that the moisture content, weight, and shape of Sichuan peppers improved progressively, peaking at the LSE harvest period. Throughout maturation, the energy content of the Sichuan pepper remained stable. Polyphenols and flavonoids, indicators of antioxidant capacity, increased in later harvest periods. A total of 18 amino acids were detected in Sichuan pepper. Proline was the most abundant amino acid, followed by serine, arginine, and glutamic acid, accounting for 83% of the total amino acids. Based on the taste threshold values of amino acids, a taste activity value (TAV) analysis of the amino acids was conducted. The TAV analysis of arginine and glutamic acid were greater than 1, indicating their significant contribution to the bitterness and umami taste, respectively. Through the principal component analysis of the electronic tongue, it was found that Sichuan pepper picked in late July (LSA stage) had a significant difference from that picked in September (LSD and LSE stages), while the difference in taste characteristics between Sichuan pepper in early September and late September was relatively small. Terpenes were the primary volatile compounds, and the number of compounds increased as the harvest period was delayed. PLS-DA analysis revealed that D-limonene had the highest VIP value, indicating its significant contribution to the overall odour of Sichuan pepper, and thus can serve as an indicator for assessing the maturity of Sichuan pepper. This study offers valuable insights for optimising the harvesting period of Sichuan pepper and serves as a theoretical reference for enhancing the development of the seasoning industry.

## 1. Introduction

Sichuan pepper (*Zanthoxylum bungeanum* Maxim) belongs to the genus *Zanthoxylum* in the family Rutaceae. It is native to southwest China, primarily cultivated and consumed in China, and usually harvested from July to September [1]. Sichuan pepper growth can be divided into six stages: budding, flowering, fruiting, swelling, oil gland growth, and harvesting. The colour of Sichuan pepper evolves during the growth period, starting as green, then turning to yellow, followed by red, and finally to dark red. *Zanthoxylum bungeanum* Maxim exhibits a more pronounced aroma than other species of the genus *Zanthoxylum* [2]. It is also rich in chemicals, such as volatile oils [3], terpenes [4], amides [5], flavonoids [5], and alkaloids [5], which contribute to its distinctive flavour and also provide rich nutritional contents. Sichuan pepper is one of the eight traditional Chinese spices. When combined with chili peppers, it creates a unique, numbing, and flavourful experience [6]. Sichuan pepper serves not only as a culinary spice and condiment in the dining and food industries but also possesses various biological functions, such as anti-inflammatory, antibacterial, anticancer, analgesic, and antiviral properties [7,8,9]. The antioxidant properties of Sichuan pepper are primarily related to the polyphenols, flavonoids, peptides, and polysaccharides [10].

Current research on Sichuan pepper primarily focuses on analysing the quality differences among various origins and varieties, bitterness constituents, and the formation mechanism of its numbing sensation [3,4,5,6,7,8,9]. Research on different varieties of Sichuan pepper indicates that *Zanthoxylum bungeanum* Maxim and *Zanthoxylum schinifolium* possess distinct primary aromatic components. The former contains more terpenes and esters and fewer alcohols than the latter [11,12]. Research on the numbing sensation suggests that numbness is a key sensory characteristic of Sichuan peppers, with sanshool as the primary causative substance. However, because of their highly unsaturated conjugated diene structures, these substances are unstable and prone to oxidation and decomposition reactions [13,14]. In addition, Yang et al. (2022) revealed that acid amide, phenols, flavonoids, and coumarin might attribute to the bitterness of Sichuan pepper [15].

Generally, multiple factors affect the quality of Sichuan peppers, including climate, soil conditions, maturity, harvesting time, and storage conditions [11,16]. This study was designed from the perspective of the impact of the harvest period on the quality of Sichuan pepper. The 24 solar terms, known as the fifth invention after China’s Four Major Inventions [17], are a traditional Chinese method for calculating time. They are divided into 24 solar terms according to the position of the sun in the ecliptic, with each marking the beginning or end of a season [18]. The 24 solar terms are pivotal in mirroring climatic shifts, thereby holding significant importance in the areas of agriculture, culinary processing, and habitation [19]. Currently, the application of the 24 solar terms in agricultural practices has primarily been based on folklore proverbs rather than scientific research. For instance, it is commonly suggested that “Wheat hastens as the Vernal Equinox passes, day and night bustling with activity” and “Plant melons at Qingming, carts and boats laden with harvest”. However, these proverbs are not empirically supported by scientific research. Only limited research on the 24 solar terms has been reported, which revealed the flavour profile variations of Huangjiu brewed under different solar terms [20]. To the best of our knowledge, this is the first study to investigate the physicochemical and flavour properties of Sichuan pepper harvested at different periods based on the 24 solar terms.

This study compared the growth characteristics, physicochemical components, and flavour properties of Sichuan peppers (LSA, LSB, LSC, LSD, LSE) harvested at five selected time points within solar terms between late July and late September. Moisture content, thousand-grain weight, fruit shape index, nutritional and energy content, total phenolic content, total flavonoid content, and antioxidant properties of Sichuan pepper harvested at different periods were assessed. The colour change was analysed using a colour difference meter. The amino acid content was analysed, and those contributing most significantly to the overall taste were identified by taste activity value (TAV) analysis. Differences in taste properties among the samples were further analysed using e-tongue combined with principal component analysis. Finally, gas chromatography (GC)–mass spectroscopy (MS) was used to examine the differences in volatile compounds. This study provides a theoretical basis for scientifically determining the optimal harvest period and improving the harvest quality of Sichuan pepper.

## 2. Materials and Methods

### 2.1. Materials, Chemicals, and Equipment

Samples were collected from Sichuan peppers in Hui Li, Liangshan, Sichuan Province, China, in 2022, during five traditional solar terms based on the 24 solar terms: Greater Heat, Beginning of Autumn, End of Heat, White Dew, and Autumnal Equinox. The corresponding Gregorian calendar dates for the harvests were 24 July, 7 August, 22 August, 6 September, and 21 September 2022. Fresh Sichuan peppers were air dried, then the seeds were removed and stored at 4 °C. The pepper samples were labelled as LSA, LSB, LSC, LSD, and LSE according to their harvesting period, where LSA corresponds to samples harvested on 24 July, LSB on August 7, LSC on 22 August, LSD on 6 September, and LSE on 21 September (Table 1).

The reference substances rutin (purity 98%), gallic acid (purity 98%), 2,4,6-tris(2-pyridyl)-1,3,5-triazine (TPTZ), Folin–Ciocalteu’s phenol reagent, anhydrous sodium carbonate, and acetate buffer, all of analytical grade, were purchased from Shanghai Aladdin Biochemical Technology Co., Ltd. (Shanghai, China); 2,2-diphenyl-1-picrylhydrazyl (DPPH), methanol, and petroleum ether were purchased from Beijing Coolaber Technology Co., Ltd. (Beijing, China). Trolox (purity 98%) was purchased from Junkehongchuang (Beijing) Biotechnology Co., Ltd. (Beijing, China). Sodium nitrite (analytical grade) was purchased from Xilong Scientific Co., Ltd. (Shantou, China) Nitric acid and sodium hydroxide, both of analytical grade, were purchased from Tianjin Comio Chemical Reagent Co., Ltd. (Tianjin, China). Ferric chloride of analytical grade was purchased from Chengdu Kelong Chemical Reagent Factory (Chengdu, China).

The following equipment was also used: XK30-A6 electronic platform scale, Shanghai Precision Instrument Company; Mitutoyo Vernier callipers (accuracy 0.05 mm), Wuxi Sierte Technology Co., Ltd. (Wuxi, China); SG9200T ultrasonic cleaner, Shanghai Guante Ultrasonic Instrument Co., Ltd. (Shanghai, China); ultraviolet–visible spectrophotometer, Beijing Lebo Tyke Instrument Co., Ltd. (Beijing, China); NH310 precision colour difference meter, Shenzhen Sanenchi Technology Co., Ltd. (Shenzhen, China); TMS-Pro food physical analyser, FTC Corporation (Washington, DC, USA); DS-1 high-speed crusher, Shanghai Specimen Model Factory (Shanghai, China); CA-HM type food calorie content detector, JWP Company of Japan (Tokyo, Japan); S-433D automatic amino acid analyser, Sykam, Germany; FOX 4000 electronic nose, Alpha MOS (Toulouse, France); Astree electronic tongue, Alpha MOS, France; GC-MS-QP2010 Plus gas chromatograph–mass spectrometer, SHIMADZU Company of Japan (Kyoto, Japan). Other instruments are common laboratory equipment, such as a portable pH meter, Shanghai Instrument & Electrical Scientific Instrument Co., Ltd. (Shanghai, China).

### 2.2. Determination of Growth Characteristics

#### 2.2.1. Determination of Moisture Content

Sichuan pepper samples were ground, and 5 g (accuracy of 0.0001 g) was weighed and placed in a weighing bottle. The bottle was then covered, weighed precisely, and placed in a drying oven (GZX-9070MBE, Shanghai Boxun Industrial Co., Ltd. (Shanghai, China). Medical equipment factory, China) at 105 °C, with the cap propped at an angle with respect to the side of the bottle. After drying for 2 to 4 h, the bottle was capped, removed from the oven, and placed in a desiccator to cool down for 30 min, and reweighed. The sample was then returned to the oven set between 101 °C and 105 °C for 1 h, removed, placed in a desiccator to cool down for 30 min, and weighed again [21].

$$W=\frac{m_{1}-m_{2}}{m_{1}-m_{0}}\times 100$$

where

*W*—moisture matter (by mass fraction),%;

*m*_0_—mass of flat bottom box, in grams (g);

*m*_1_—mass of flat bottom box and sample before drying, in grams (g);

*m*_2_—mass of flat bottom box and dried sample, in grams (g).

#### 2.2.2. Determination of Thousand-Grain Weight

Samples were divided using the quartering method to approximate a specified weight (5 g). The weight (W) was accurately measured, and the grains were counted to obtain m. This procedure was repeated four times. The thousand-grain weight (g per 1000 grains) was calculated as (W/m) × 1000 [22].

#### 2.2.3. Determination of Fruit Shape Index

The diameters were measured using Vernier callipers. The longitudinal (mm) and transverse diameters (mm) of Sichuan pepper fruits were measured, and the fruit shape index was calculated as the longitudinal diameter divided by the transverse diameter [23].

### 2.3. Determination of Colour Difference

A colorimeter (NR200+, 3 nh Intelligent Technology Co., Ltd., Guangzhou, China) was used to determine the colour of Sichuan peppers harvested at different periods. Three measurements were taken per sample at different positions ranging from the centre to the edge. These measurements included L* (lightness), a* (red-green hue), and b* (yellow-blue hue) values. Smaller L* values indicated darker colours, smaller a* values indicated a greener hue, and smaller b* values suggested bluer hues [24].

### 2.4. Determination of Total Phenolic Content, Total Flavonoid Content, and Antioxidant Activities

#### 2.4.1. Determination of Total Phenolic Content

The Folin–Ciocalteu method was employed for polyphenol measurement. Approximately 300 μL of sample (1 mg/mL extraction) was added to 1 mL of FC reagent (1 mol/mL), and the mixture was left for 3 min before adding 0.8 mL of 7.5% (*w*/*v*) sodium carbonate. A standard curve was plotted using gallic acid as the reference standard within the concentration range of 0.0005 to 0.0050 mg/mL. The absorbance was measured at 765 nm using a UV–vis spectrophotometer (UV3600, Shimadzu, Kyoto, Japan) after 2 h. A standard curve with gallic acid as the standard was plotted, with the regression equation of the standard curve being Y_1_ = 0.1708X_1_ − 0.0025, R^2^ = 0.9993 (where Y_1_ represents absorbance and X_1_ is the concentration of gallic acid in mg/mL). The total phenolic content (mg GAE/g) was calculated using gallic acid [25].

#### 2.4.2. Determination of Total Flavonoids

Briefly, 3 g of sample was ground using liquid nitrogen and subsequently subjected to ultrasonic extraction with 30 mL of 80% methanol (*v*/*v*) at 400 W and 25 °C for 20 min. Following filtration, the residues underwent two additional extractions. The combined extracts were then vacuum concentrated to 10 mL and defatted three times with petroleum ether (1:2 *v*/*v*), and the hydrophilic phase was adjusted to a final volume of 15 mL with 80% methanol. Then, 0.15 mL of the sample solution was pipetted, followed by addition of 0.3 mL of 5% NaNO_2_ solution and shaking. After incubation for 6 min, 0.3 mL of 10% Al(NO_3_)_3_ solution was added and shaken again. After another 6 h of incubation, 4.0 mL of 4% NaOH solution was added, and the volume of the reaction solution was made up to 10 mL with 40% ethanol and left to stand for 15 min. The absorbance value was measured at 510 nm using a UV–vis spectrophotometer (UV3600, Shimadzu, Kyoto, Japan). A standard curve within the range of 0.01120.0914 mg/mL was constructed using rutin as the standard substance. The linear regression equation was obtained as Y = 13.876X − 0.005, with R^2^ = 0.9994 [26].

#### 2.4.3. Determination of DPPH Free Radical-Scavenging Activity

Three-gram samples were mixed with 30 mL of ethanol and incubated at 60 °C in a shaking water bath for 3 h. The mixture was then filtered through filter paper, and 2 mL of the filtrate was mixed with 2 mL of 0.4 mM methanolic DPPH solution. This mixture was kept in the dark for 30 min, with ethanol used as a control. The absorbance was measured at 517 nm using a UV–vis spectrophotometer (UV3600, Shimadzu, Kyoto, Japan), with 100% methanol as the blank. The DPPH radical-scavenging activity was calculated using a specific equation [25].

$$DPPH\;radical-scavenging\;activity\left(\%\right)=1-\frac{Absorbance\;of\;sample\;assay\;}{Absorbance\;of\;control\;assay}\times 100\%$$

#### 2.4.4. Determination of the FRAP Free Radical-Scavenging Activity

FRAP reagent was prepared by mixing 10 mmol/L TPTZ, 20 mmol/L FeCl^3^, and 300 mmol/L acetate buffer at a volume ratio of 1:1:10. The sample was diluted with 40% ethanol to 2 mg/mL. Then, 0.2 mL of the sample solution was mixed with FRAP reagent preheated to 37 °C. The mixture was reacted in a water bath at 37 °C for 10 min. The absorption value was measured at 593 nm, with 40% ethanol as the blank control. A standard curve within 0.01–0.1 mg/mL was constructed with Trolox solution as the positive control. The linear regression equation was determined as Y = 17.556X + 0.3428 with R^2^ = 0.9991. The total reduction capacity of samples was calculated [27].

### 2.5. Determination of Amino Acids

A 1 g sample of Sichuan pepper was collected and combined with 8 mL of 7% sulfosalicylic acid. The mixture was ultrasonicated for 30 min at 40 °C and left to stand for 1 h. Subsequently, the pH was adjusted to 2.2. The mixture was then centrifuged (TGL-18, Shuke Instrument Co., Ltd., Chengdu, China) at 10,000 rpm for 10 min. The supernatant was collected and analysed using an automatic amino acid analyser (S433D, Sykam, Munich, Germany). The chromatographic column used was LCAK07/Li (150 mm × 4.6 mm) with a sulfonic acid-based strong acid cation-exchange resin separation column. The injection volume was 50 µL; detection wavelengths were set at 570 nm and 440 nm; the reactor temperature was maintained at 130 °C and the column temperature at 58 °C [28].

### 2.6. Protein, Lipid, Carbohydrate, and Energy Determination

Protein, fat, carbohydrate, and energy determination was performed using a CA-HM Food Calorimetric Component Analyser (JWP, Tokyo, Japan). The analytical procedure was performed with each sample undergoing three measurements. The values reported for each parameter are the averages of these independent measurements, ensuring reproducibility of the results [29].

### 2.7. E-Tongue Analysis

E-tongue analysis was conducted using the α-ASTREE instrument (Alpha MOS, Toulouse, France) with a sixteen autosampler carousel for sample handling. The instrument was equipped with specific sensors for sourness, umami, and saltiness, corresponding to the AHS, NMS, and CTS sensors, respectively. In addition, there were a total of 7 sensors, including CPS, ANS, SCS, PKS, etc. Ag/AgCl was selected as the reference electrode. A 10 g sample was taken, crushed, wrapped in gauze, and added to 150 g of distilled water. The mixture was boiled on an induction cooker at 1200 W, with the power adjusted to 800 W after boiling, and maintained for 20 min, followed by 30 min of ultrasonication at 400 W. After filtration, 80 mL of the filtrate was transferred to a specialised electronic tongue flask for measurement. The supernatant was then used for analysis. The data acquisition time was 120 s, with a sampling interval of 1.0 s and a sampling delay of 0 s. The stirring speed was set at 1 r/s. Each sample was measured 8 times in replicates, and the stable values from the last 3 measurements were taken as the detection results [30].

### 2.8. HS-GC-MS Analysis

#### 2.8.1. GC Conditions

A 0.5 g sample was accurately weighed, placed in a 10 mL headspace vial, sealed, and labelled. The chromatographic column used was an Rtx-5MS (30 m × 0.25 mm, 0.25 μm) (Waltham, MA, USA), with a column temperature set at 40 °C and an injection temperature of 270 °C. The sample was injected at a pressure of 49.5 kPa, column flow rate of 1.0 mL/min, and split ratio of 3.0. The initial temperature was maintained at 40 °C for 5 min, then increased to 150 °C at a rate of 5 °C/min, maintained for 2 min, followed by an increase to 280 °C at a rate of 10 °C/min, and finally maintained for 3 min [31].

#### 2.8.2. MS Conditions

The mass spectroscopy conditions included using an electron ionisation source, with an ion source temperature of 200 °C and an interface temperature of 220 °C. The solvent delay time was 0.1 min. MS acquisition started at 0.2 min and ended at 45 min at intervals of 0.5 s. The mass scan range was set to 30–500 *m*/*z*. Silicon-containing substances that were lost during column separation were excluded. Compounds with forward and reverse match scores above 800 (out of a maximum of 999) were selected. Qualitative analysis was conducted using the NIST (2011) spectral libraries alongside manually interpreted mass spectra. The relative content is calculated based on the relative peak area [31].

### 2.9. Data Processing

All experiments were conducted in three replicates and expressed as mean ± standard deviation (SD), which was assessed using one-way ANOVA with Turky’s test, and mean values were thought significantly different at *p* < 0.05, using SPSS 23.0 Statistics (IBM Corp., Armonk, NY, USA). Graph and principal component analyses (PCAs) were performed using Origin 2023 (Alpha MOS, Toulouse, France). Partial least squares discriminant analysis (PLS-DA) and VIP were carried out using SIMCA 14.1 software (Umetrics, Umea, Sweden).

## 3. Results and Discussion

### 3.1. Growth Characteristics of Sichuan Peppers at Different Harvesting Periods

Differences in moisture content, thousand-grain weight, fruit shape index, and nutritional components were noted among Sichuan peppers harvested at different periods (Table 2). The moisture content of the samples increased steadily with harvesting duration, maintaining stability during the LSB to LSC periods at approximately 10%, indicating the stable water accumulation of Sichuan pepper at these periods. The thousand-grain weight of the samples progressively increased with harvesting duration, reaching a maximum of 2.57 g during the LSE period. This increase was likely due to the continuous expansion of oil glands on the surface of Sichuan peppers and the accumulation of contents within [32], which can bring implication on the potentially enriched flavour of the Sichuan peppers during this harvest period. The Sichuan pepper fruit shape index, calculated as the ratio of the longitudinal diameter to the transverse diameter, increased with harvesting time, peaking at 1.19 during the LSE period, suggesting optimal external appearance. Throughout the harvesting periods, the energy content of the samples ranged between 334 and 360 kcal/100 g, with minor variations. The protein content approximately ranged from 6 to 10 g/100 g, fat content from 5 to 9.5 g/100 g, and carbohydrate content from 57 to 66 g/100 g. The energy difference of Sichuan peppers harvested at different periods was small, and the change law of carbohydrate, protein, and fat content was not obvious, which needs to be further studied.

### 3.2. Analysis of Flavonoids, Total Phenols, and Antioxidant Activity in Sichuan Pepper at Different Harvesting Periods

**Figure 1:**
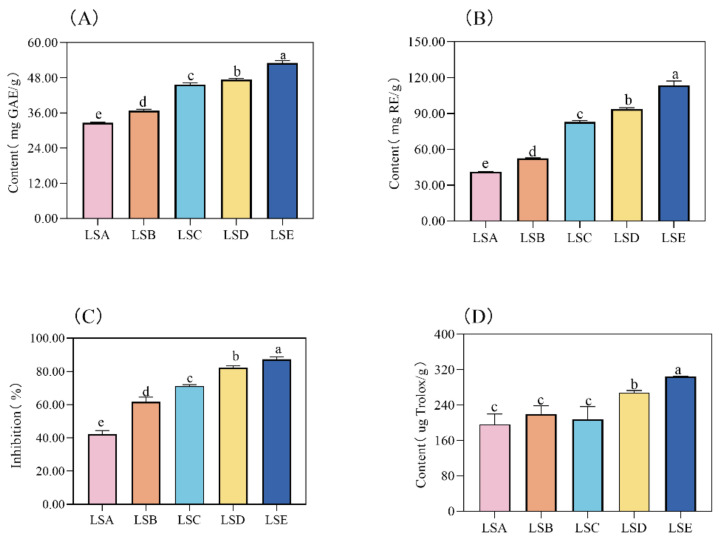
Variations in total phenols (**A**), flavonoids (**B**), DPPH free radical-scavenging rate (**C**), and Ferric reducing antioxidant power (**D**) in Sichuan pepper at different harvesting periods. Values marked by the same lowercase superscript letters (from “a” to “e”) denote statistically significant differences (*p* < 0.05).

Phenolic and flavonoid compounds in Sichuan pepper possess diverse biological activities, such as antioxidant [3], antibacterial, anti-aging [1], and anti-cardiovascular properties [2], which are beneficial for human health. Studies have reported that the flavonoid content in Sichuan pepper is relatively high, accounting for approximately 11.2%, which is thought to help the plant resist biotic and abiotic environmental stress, often contributing to a bitter or astringent taste [16,33]. Phuyal et al. [26] studied flavonoids in the bark, fruit, and seeds of Sichuan pepper and found that the flavonoid content and antioxidant activity in the fruit were higher than those in the bark, with the lowest content found in the seeds. Interestingly, active components of *Zanthoxylum armatum* are related to its growth environment, with total polyphenols, total flavonoids, and antioxidant activity varying depending on the environment. Karmakar et al. [34] identified three flavonoids from Sichuan pepper leaves: rutin, quercetin, and myricetin, and demonstrated that these extracts possessed robust cytotoxicity and antitumor activity. In the current study, a consistent and significant increase (*p* < 0.05) in total phenols and flavonoids in Sichuan pepper throughout the maturation process was observed (Figure 1A,B), peaking at 53.04 mg/g and 113.46 mg/g, respectively, during the LSE period. This observation suggests heightened physiological activity in Sichuan pepper during the LSE period (mid-to-late September). This observation is consistent with previous findings [35] of an increase in flavonoid and total phenol contents in Sichuan pepper during the maturation period. Anthocyanin compounds in phenolic and flavonoid substances influence the colour of Sichuan pepper pericarp [1,2]. With the increase in total phenols and flavonoids, the colour of Sichuan pepper pericarp changes from light red to dark red. Total phenols and flavonoids also contribute to taste profiles, such as sweetness, sourness, and bitterness [36]. High total phenol and flavonoid contents are linked to the bitterness of the pericarp of Sichuan pepper [15]. In summary, Sichuan peppers harvested in mid-to-late September had darker colours and stronger physiological activity but may have a slightly bitter taste.

Excessive accumulation of free radicals in the body can lead to the occurrence of aging and diseases. The intake of antioxidants alleviates the damage caused by oxidative stress, as antioxidants effectively scavenge reactive oxygen and reduce oxidative stress. The DPPH method is the most commonly used method for evaluating antioxidant activity [37]. The highest antioxidant capacity was observed during the LSE period. During this time, the levels of phenolics and flavonoids increased. This correlation suggests that as the harvesting time is prolonged within the maturation period, the antioxidant properties of Sichuan peppers improve. It was observed that the DPPH free radical-scavenging rate and Ferric reducing antioxidant power were consistent with those of the total phenols and flavonoids (Figure 1C,D).

### 3.3. Analysis of Colour Characteristics of Sichuan Pepper at Different Harvesting Periods

**Figure 2:**
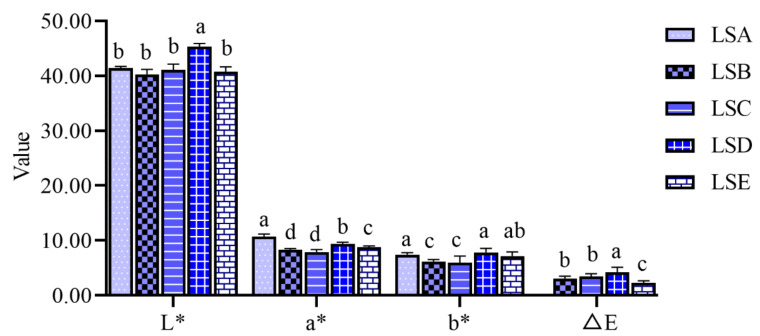
Colour differences of Sichuan peppers at different harvesting periods. Values marked by the same lowercase superscript letters (from “a” to “d”) within bars denote statistically significant differences (*p* < 0.05).

The colour of Sichuan pepper results from the accumulation of pigments during the growth process. The colour primarily results from the breakdown of chlorophyll and carotenoids, along with the accumulation of anthocyanins and flavonoids [38]. The L value initially increased and then decreased with the extension of harvesting time, peaking during the LSD period, which was significantly different from that of Sichuan peppers harvested in other periods (*p* < 0.05) (Figure 2). This observation suggests that Sichuan peppers harvested during the LSD period had the highest brightness. Both a* and b* values were positive, indicating that the colour of the Sichuan peppers leaned towards red and yellow hues, with the a* value decreasing over the harvesting period, signifying a change in Sichuan pepper colour from red to dark red at the later harvesting periods. Earlier studies have reported that the colour of Sichuan pepper undergoes several changes during the maturation process: pale red, light red, red, and dark red, with the light-red phase being critical for the formation of the Sichuan pepper colour [38]. Anthocyanins contribute to the formation of red, purple, and blue colours in many plant organs [39]. △E represented the total colour difference between samples at various periods and the LSA period. As shown in Figure 2, the colour difference between the LSD period and LSA period was the largest, while the colour difference between the LSE period and LSA period was the smallest.

### 3.4. Analysis of Free Amino Acids in Sichuan Pepper at Different Harvesting Periods

Amino acids are essential components of food, constitute the basic units of proteins, and play a significant role in the nutritional value and flavour of Sichuan pepper [40]. Across the harvesting periods, eighteen free amino acids in samples were detected, including seven essential and eleven non-essential amino acids (Table 3). The essential amino acids were threonine, valine, tryptophan, isoleucine, leucine, phenylalanine, and lysine and the non-essential amino acids were aspartic acid, serine, glutamic acid, glycine, alanine, cysteine, tyrosine, histidine, arginine, proline, and asparagine. Total free amino acid content ranged from 799.55 to 1199.22 mg/100 g, increasing from LSA to LSC, indicating that amino acid-related nutrients were further synthesised as the growth and maturation times increased. However, a decrease was noticed in the LSD period samples, but then free amino acid content peaked in the LSE period. The content of amino acids not only reflects the nutritional value of Sichuan pepper but also serves as the substrate for the further generation of flavour compounds. Free amino acids provide taste characteristics, such as umaminess, sweetness, and bitterness, significantly enriching the taste profile of food [40].

Based on taste characteristics, the tasting components of amino acids are categorised as umami (glutamic acid, aspartic acid, and asparagine), sweet (proline, serine, threonine, histidine, alanine, and glycine), bitter (arginine, leucine, tryptophan, valine, isoleucine, lysine), and aromatic (cysteine, phenylalanine, and tyrosine) [41]. This study observed variations in amino acids of Sichuan pepper across different harvesting periods, with sweet-tasting amino acids dominating initially, followed by bitter, umami, and finally aromatic amino acids. The LSD period exhibited the highest contents of aromatic, bitter, and umami amino acids, at 19.38 mg/100 g, 232.07 mg/100 g, and 159.44 mg/100 g, respectively, while sweet amino acids were higher in the LSC period. These observations suggest varying contributions of different taste-contributing amino acids at different stages of maturity. During Sichuan pepper fruit growth, large amounts of nitrogen compounds synthesised in branches and leaves enhance the accumulation of amino acids in the fruit [42]. Proline was the most abundant amino acid, followed by serine, arginine, and glutamic acid. Together, these four amino acids constituted 83% of the total amino acid content in Sichuan pepper. This observation contrasts with a previous study that identified lysine and tryptophan as abundant amino acids in Sichuan peppers at different periods, which may be due to differences in Sichuan pepper varieties, tree age, and growth environment of Sichuan peppers and other factors [43]. Proline and serine contribute to the formation of a refreshing sweet taste in Sichuan pepper, similar to that of lemons and citrus. Glutamic acid, which is abundant in cereal proteins, provides umami-like characteristics and is essential for protein metabolism and many vital biochemical reactions in organisms [40,44]. In contrast, arginine is linked to the bitter taste of Sichuan peppers [45]. Hence, the high accumulation of these four free amino acids could be a significant source of the unique flavour of mature Sichuan peppers in Hui Li. Valine and leucine are precursors for the synthesis of the nitrogenous part of sanshool, the compound responsible for the numbing sensation in Sichuan pepper, and their levels significantly influence the numbing taste characteristics of Sichuan pepper [46]. Their contents varied between 3% and 9% throughout the harvesting period, with the highest concentration observed in the LSB period (early August). The leucine content remained at approximately 2% during the entire harvesting period, peaking in the LSD period (early September). These findings suggest that Sichuan peppers harvested during these two periods may have better numbing properties. The light, temperature conditions, water, and soil nutrients at different harvest periods may affect the metabolic activities of Sichuan pepper, thereby influencing the synthesis and accumulation of amino acids in pepper [16]. In addition, during the growth period, Sichuan pepper may require more amino acids for protein synthesis, while during the mature period, more amino acids may be used for secondary metabolism [47].

### 3.5. TAV Analysis of Key Amino Acids in Sichuan Pepper at Different Harvesting Periods

**Figure 3:**
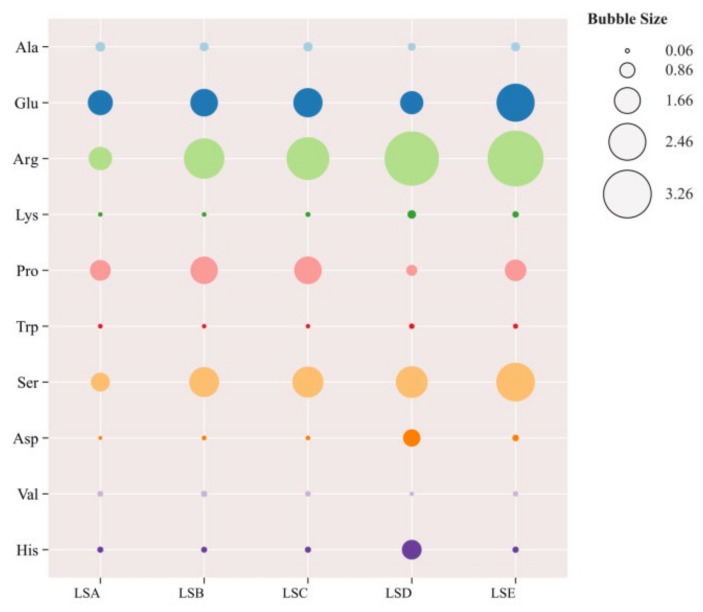
Visualization of amino acid TAV values using a bubble chart (https://www.omicshare.com/). Different colors in the figure represent different amino acids.

Because of the different taste thresholds of each amino acid, simply analysing their content may not accurately reflect their contribution to the overall taste of Sichuan peppers. Therefore, TAV analysis was performed to evaluate their taste contribution [4]. TAV is calculated by dividing the concentration of the amino acid by its taste threshold. A TAV greater than one indicates a significant taste contribution, with higher values indicating a more pronounced taste effect, while a TAV below one suggests a smaller contribution and a less noticeable taste effect [48]. As shown in Figure 3, arginine, lysine, and valine exhibited the highest TAVs among the bitter amino acids. Arginine consistently showed TAVs exceeding 1 across all harvesting periods, peaking at 3.86 in the LSE period, indicating its significant contribution to the bitterness of Sichuan pepper. Studies have also suggested that the bitterness of Sichuan pepper intensifies as the fruit matures [45]. For sweet amino acids, proline, threonine, serine, and histidine contributed to the sweetness of Sichuan pepper. Both proline and serine exhibited TAVs exceeding one across all samples, with the highest values noticed during the LSC and LSE periods, respectively, indicating their contribution to the sweetness during these periods. For umami amino acids, glutamic acid had a TAV greater than 1 in all samples, with the highest value of 2.56 in the LSE period, indicating that glutamic acid contributed most significantly to the umami taste of Sichuan pepper, which gradually intensified as the Sichuan pepper matured in late September.

### 3.6. Analysis of Taste Characteristics of Sichuan Pepper at Different Harvesting Periods Based on E-Tongue Analysis

**Figure 4:**
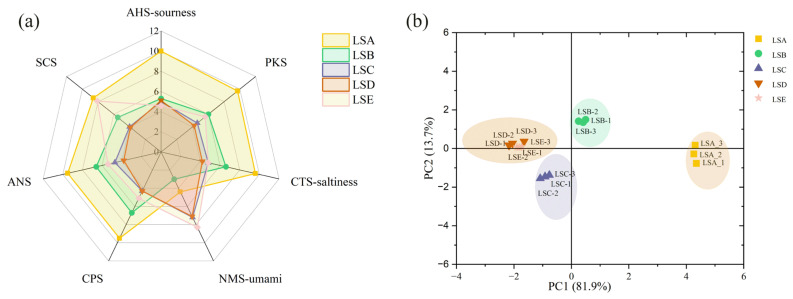
Taste characteristics of Sichuan pepper at different harvesting periods analysed by e-tongue. (**a**) Radar chart; (**b**) principal component analysis (PCA).

The e-tongue, an intelligent sensory technology, uses artificial lipid membrane sensors, working on principles similar to those of human taste buds, to objectively and digitally evaluate basic taste sensations in food, such as sourness, bitterness, astringency, saltiness, and umami [49]. Sichuan pepper samples from different harvesting periods showed varied response values across sensors, indicating differences in the overall taste characteristics of Sichuan peppers (Figure 4a). Samples from the LSC to LSE periods exhibited higher values for umami taste sensing than those from LSA to LSB periods, which had lower values. This observation suggests an intensification of umami characteristics in Sichuan pepper with extended harvesting time, which is consistent with the amino acid trend. Research indicates that various factors influence the taste of Sichuan pepper. For example, studies have shown that Sichuan peppers from different regions exhibit significant differences in taste characteristics (sourness, saltiness, umami) [43].

To further analyse the taste characteristics of the samples, principal component analysis (PCA) was used to assess the differences in taste characteristics of Sichuan pepper at various harvesting periods [50]. PC1 accounted for 81.9% and PC2 for 13.7%, with a cumulative contribution of over 80%, suggesting the high reliability of the data (Figure 4b). Samples were clustered into four groups, with LSA as one group, LSB as another, LSC as a third, and both LSD and LSE as the fourth group. On the PC1 axis, LSA distinctly differentiated from LSD and LSE, indicating significant differences in taste between Sichuan peppers harvested in mid-to-late July (LSA) and those harvested in September (LSD and LSE). As Sichuan peppers matured, taste differences reduced, particularly between the early and late September harvests.

### 3.7. Analysis of Volatile Compounds in Sichuan Pepper at Different Harvest Periods Through GC-MS

**Figure 5:**
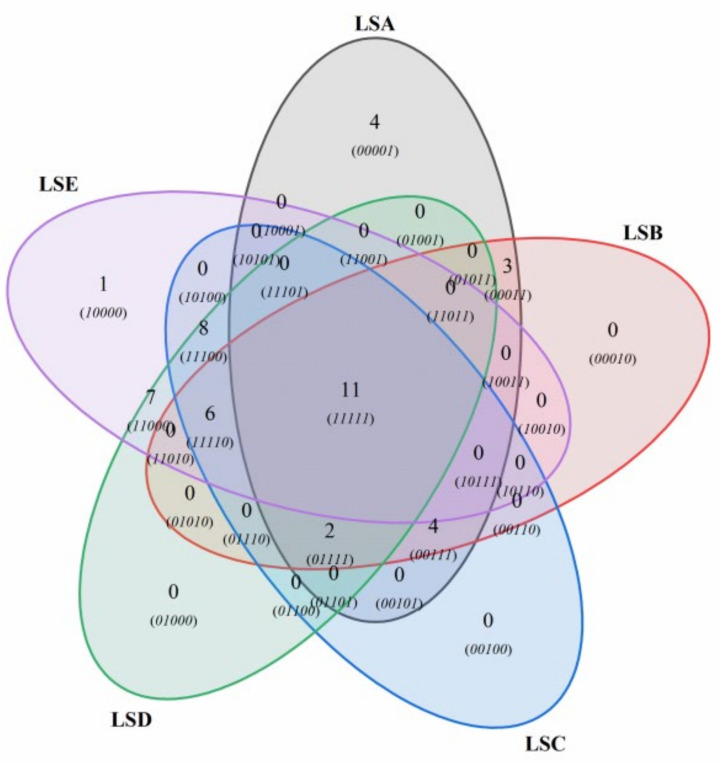
Volatile compound analysis in Sichuan pepper at different harvest periods.

The volatile compounds present in Sichuan peppers at different harvest time points were analysed using GC-MS (Figure 5). A total of 46 volatile compounds were detected across all samples. These compounds included four alcohols, seven aldehydes, twenty-two terpenes, six alkanes, and five esters. The number of identified compounds varied across the samples, with LSA, LSB, LSC, LSD, and LSE containing 24, 26, 31, 34, and 33 types of compounds, respectively. Terpenes emerged as the predominant flavour compounds, showing significant variability across the different harvesting periods. Their contents across the samples LSA, LSB, LSC, LSD, and LSE were 49.08%, 79.2%, 94.12%, 85.59%, and 89.99%, respectively. Among all samples, 11 common compounds were identified, with D-limonene and β-pinene being the primary. Their concentrations initially increased and then decreased as the Sichuan peppers matured.

### 3.8. Multivariate Statistical Analysis to Identify Key Volatile Compounds in Sichuan Pepper

**Figure 6:**
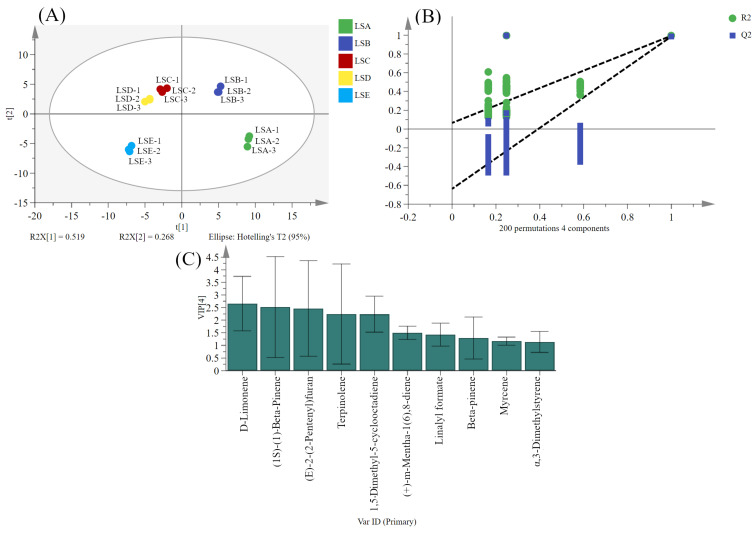
Analysis of key volatile compounds in Sichuan pepper at different harvest periods based on PLS-DA. (**A**) Score plot; (**B**) cross-validation; (**C**) compounds with a VIP > 1.

To further analyse the compounds in the samples, PLS analysis on the data obtained from GC-MS was employed next. The results revealed high repeatability and notable differences among the Sichuan peppers harvested at different time points (Figure 6A). Samples were divided into three categories based on their positioning on PC1: the first group consisted of LSA samples, the second included LSB, LSC, and LSD samples, and the third category contained LSE samples. This result indicates that the LSA and LSE samples had significant differences in overall odour, while the odour characteristics of the LSB, LSC, and LSD samples were more similar, possibly because of the continuous growth of the Sichuan pepper and the formation of more volatile substances as the harvest time progressed. To further verify the reliability of the model, a 200-times cross-validation was conducted, which revealed an R^2^ slope greater than zero and a Q^2^ intercept less than zero, indicating that the model was not overfitted and could be analysed further (Figure 6B). Key volatile compounds in the samples were screened using VIP values. Ten compounds with VIP > 1 were identified across the five different harvest periods, signifying these compounds as characteristic of Sichuan pepper samples harvested at various periods (Figure 6C). Notably, D-limonene exhibited the highest VIP value, suggesting its significant contribution to the overall odour of Sichuan pepper.

## 4. Conclusions

This study confirms the previously hypothesised variation in the physicochemical and flavour properties of Sichuan pepper harvested at different periods based on the 24 solar terms.

This study highlights that moisture content and the thousand-grain weight increased steadily, with the fruit shape index peaking in September. The total polyphenols and flavonoids rose consistently with the extension of the harvest period, enhancing the antioxidant capacity. Out of eighteen free amino acids detected, the highest total amino acid content was observed at the LSE period. Proline was the most abundant amino acid, followed by serine, arginine, and glutamate; all four together made up 83% of the total amino acid content.

The TAV calculations revealed that arginine significantly contributed to bitterness in the later maturity periods. E-tongue analysis, which employed PCA, indicated significant taste differences between the early (LSA period; late July) and late harvest periods (LSD and LSE periods; September); however, these differences gradually diminished as the harvest period was extended (less difference between LSD and LSE periods). This observation indicates that September is an appropriate month for harvesting Sichuan peppers.

GC-MS analysis demonstrated that terpenes were the main volatile compounds in Sichuan pepper across different harvest periods and D-limonene had the highest VIP value based on PLS-DA analysis, suggesting that it prominently contributes to the overall flavour and that its concentration could serve as an indicator of Sichuan pepper maturity. However, this study is limited by its single-season and single-region design. Future work should validate these findings across diverse growing conditions and integrate sensory panels to bridge instrumental measurements with consumer perception.

## Figures and Tables

**Table 1:** Environmental data of Sichuan pepper across five harvesting periods selected in this study within the 24 traditional Chinese solar terms.

Sample ID	Harvesting Time	Solar Term *	Average Highest Temperature (°C)	Average Lowest Temperature (°C)	Average Humidity (%)	Average Rainfall (mm/h)	Average Ground Wind Speed (m/s)
LSA	24 July 2022	Greater Heat	29	19	69.5	0.32	1.44
LSB	7 August 2022	Beginning of Autumn	26	18	81.0	0.53	0.81
LSC	22 August 2022	End of Heat	28	18	74.0	0.31	0.91
LSD	6 September 2022	White Dew	26	17	77.8	0.37	1.23
LSE	21 September 2022	Autumn Equinox	24	17	81.2	0.41	0.82

* The environmental data were obtained from the China Weather Historical Database (https://lishi.tianqi.com/), based on the altitude, latitude, and longitude of the Sichuan pepper cultivation location.

**Table 2:** Moisture content, thousand-grain weight, fruit shape index, and nutritional components of Sichuan peppers at different harvesting periods.

Harvest Periods	Moisture Content (%)	Thousand- Grain Weight (g)	Transverse Diameter (mm)	Longitudinal Diameter (mm)	Fruit Shape Index	Energy (kcal)	Protein (g/100 g)	Fat (g/100 g)	Carbohydrates (100/g)
LSA	9.23 ± 0.11 ^c^	1.42 ± 0.02 ^d^	3.75 ± 0.46 ^a^	4.29 ± 0.30 ^c^	0.94 ± 0.12 ^b^	337.33 ± 2.05 ^c^	9.50 ± 0.22 ^a^	5.30 ± 0.14 ^d^	64.33 ± 1.07 ^b^
LSB	10.68 ± 0.17 ^b^	1.47 ± 0.05 ^cd^	4.33 ± 0.17 ^ab^	4.56 ± 0.32 ^bc^	1.07 ± 0.10 ^ab^	352.00 ± 2.16 ^b^	7.03 ± 0.48 ^b^	6.70 ± 0.08 ^c^	66.17 ± 0.25 ^a^
LSC	10.70 ± 0.06 ^b^	1.58 ± 0.12 ^c^	4.44 ± 0.19 ^c^	4.20 ± 0.32 ^c^	1.12 ± 0.12 ^a^	326.67 ± 2.36 ^d^	7.63 ± 0.05 ^b^	5.57 ± 0.25 ^d^	60.23 ± 0.83 ^c^
LSD	10.57 ± 0.14 ^b^	1.93 ± 0.06 ^b^	4.58 ± 0.29 ^bc^	4.73 ± 0.58 ^b^	1.16 ± 0.04 a	358.67 ± 2.05 ^a^	9.97 ± 0.24 ^a^	9.47 ± 0.09 ^a^	57.90 ± 0.80 ^d^
LSE	15.89 ± 0.03 ^a^	2.57 ± 0.04 ^a^	4.08 ± 0.57 ^ab^	5.28 ± 0.34 ^a^	1.19 ± 0.09 ^a^	334.00 ± 4.90 ^c^	6.00 ± 0.45 ^c^	7.20 ± 0.33 ^b^	60.73 ± 0.90 ^c^

Data are presented as mean ± standard deviation (n = 3). Mean values sharing the same lowercase superscript letters within a column denote statistically significant differences (*p* < 0.05).

**Table 3:** Content of free amino acids in Sichuan pepper at different harvesting periods.

FAA	Taste Characteristics	Content (mg/100 g)				
		LSA	LSB	LSC	LSD	LSE
Cysteine	Aromatic	4.39 ± 0.28 ^b^	4.32 ± 0.15 ^b^	4.67 ± 0.37 ^b^	9.29 ± 0.44 ^a^	3.89 ± 0.46 ^b^
Phenylalanine		2.48 ± 0.33 ^ab^	2.83 ± 0.45 ^a^	1.91 ± 0.18 ^b^	3.52 ± 0.42 ^a^	2.59 ± 0.34 ^ab^
Tyrosine		3.77 ± 0.39 ^b^	3.09 ± 0.17 ^bc^	2.32 ± 0.52 ^c^	6.58 ± 0.42 ^a^	2.62 ± 0.04 ^c^
Arginine	Bitter	73.81 ± 0.35 ^c^	136.42 ± 1.84 ^b^	145.04 ± 5.01 ^b^	187.38 ± 6.99 ^a^	193.17 ± 1.16 ^a^
Lysine		4.84 ± 0.16 ^e^	5.57 ± 0.15 ^d^	6.79 ± 0.12 ^c^	19.52 ± 0.17 ^a^	12.09 ± 0.32 ^b^
Leucine		2.40 ± 0.14 ^ab^	2.45 ± 0.55^ab^	1.79 ± 0.28 ^b^	2.62 ± 0.28 ^a^	2.14 ± 0.29 ^ab^
Tryptophan		9.49 ± 0.06 ^c^	8.52 ± 0.22 ^d^	8.43 ± 0.38 ^d^	15.74 ± 0.27 ^a^	13.00 ± 0.25 ^b^
Valine		7.51 ± 0.61 ^b^	9.33 ± 0.29 ^a^	6.72 ± 0.24 ^bc^	3.60 ± 0.30 ^d^	6.24 ± 0.24 ^c^
Isoleucine		2.76 ± 0.14 ^a^	3.09 ± 0.21 ^a^	2.23 ± 0.26 ^a^	3.21 ± 0.85 ^a^	2.57 ± 0.36 ^a^
Proline	Sweet	385.68 ± 4.24 ^b^	533.45 ± 0.41 ^a^	535.59 ± 3.04 ^a^	174.42 ± 5.62 ^c^	406.23 ± 16.92 ^b^
Serine		172.39 ± 1.30 ^d^	293.51 ± 1.61 ^c^	306.11 ± 2.14 ^bc^	314.88 ± 5.60 ^b^	389.30 ± 13.98 ^a^
Threonine		7.16 ± 0.42 ^bc^	11.28 ± 0.87 ^a^	11.15 ± 0.88 ^a^	6.44 ± 0.46 ^c^	8.41 ± 0.89 ^b^
Histidine		4.41 ± 0.21 ^b^	4.24 ± 0.46 ^b^	4.36 ± 0.21 ^b^	24.12 ± 1.01 ^a^	4.58 ± 0.64 ^b^
Alanine		28.80 ± 0.97 ^a^	25.97 ± 1.65 ^a^	26.99 ± 0.99 ^a^	20.37 ± 0.34 ^b^	25.73 ± 3.76 ^a^
Glycine		1.71 ± 0.13 ^c^	2.28 ± 0.25 ^abc^	1.98 ± 0.17 ^bc^	3.27 ± 0.58 ^a^	2.68 ± 0.36 ^ab^
Glutamic Acid	Umami	48.03 ± 0.07 ^c^	53.72 ± 0.33 ^b^	57.35 ± 2.76 ^b^	43.99 ± 1.74 ^d^	76.86 ± 0.14 ^a^
Aspartic Acid		6.10 ± 0.08 ^d^	11.73 ± 0.32 ^c^	10.86 ± 0.98 ^c^	103.84 ± 1.45 ^a^	25.35 ± 4.11 ^b^
Asparagine		13.83 ± 4.93 ^bc^	24.50 ± 0.82 ^a^	21.12 ± 1.37 ^ab^	11.62 ± 1.57 ^c^	21.77 ± 1.86 ^ab^
Sum Content (Aromatic)	-	10.64 ± 0.41 ^b^	10.25 ± 0.64 ^b^	8.89 ± 0.92 ^b^	19.38 ± 0.93 ^a^	9.10 ± 0.54 ^b^
Sum Content (Bitter)	-	100.81 ± 0.80 ^c^	165.38 ± 2.07 ^b^	170.99 ± 5.63 ^b^	232.07 ± 5.85 ^a^	229.21 ± 1.55 ^a^
Sum Content (Sweet)	-	600.15 ± 3.84 ^c^	870.74 ± 3.73 ^a^	886.18 ± 5.26 ^a^	543.49 ± 1.46 ^d^	836.93 ± 24.60 ^b^
Sum Content (Umami)	-	67.95 ± 4.80 ^d^	89.95 ± 0.83 ^c^	89.33 ± 3.40 ^c^	159.44 ± 2.76 ^a^	123.98 ± 5.58 ^b^
TA	-	779.55 ± 2.10 ^d^	1136.31 ± 2.92 ^b^	1155.39 ± 10.22 ^b^	954.39 ± 7.67 ^c^	1199.22 ± 21.94 ^a^

Values marked by the same lowercase superscript letters (from “a” to “e”) within a column denote statistically significant differences (*p* < 0.05). TA stands for total amino acid.

## Data Availability

The original contributions presented in this study are included in the article/Supplementary Materials. Further inquiries can be directed to the corresponding authors.

## Supplementary Materials

The following supporting information can be downloaded at: https://www.mdpi.com/article/10.3390/foods14071155/s1, Table S1. The relative contents of volatile substances in Sichuan pepper at different harvest periods.

## Abbreviations

The following abbreviations are used in this manuscript:

GC	gas chromatography
MS	mass spectroscopy
PLS-DA	partial least squares discriminant analysis
PCA	principal component analysis
TA	total amino acid
TAV	taste activity value
VIP	variable importance in projection
